# Delivery platforms for *in vivo* CAR-T engineering

**DOI:** 10.3389/fimmu.2026.1841160

**Published:** 2026-08-04

**Authors:** He Wang, Guangmei Qiao, Ming Ma, Hailong Zhao, Lin Luo, Ting Liu

**Affiliations:** 1 Clinical Laboratory, Second Affiliated Hospital of Dalian Medical University, Dalian, China; 2 Department of General Surgery, Huangyuan County People’s Hospital, Xining, China

**Keywords:** *in situ* reprogramming, *in vivo* CAR-T engineering, mRNA-LNP, next-generation cellular immunotherapy, targeted delivery vectors

## Abstract

Ex vivo CAR-T changed cancer care, but its made-to-order manufacturing keeps too many patients waiting. *In vivo* CAR-T takes a different path: deliver the genetic blueprint to a patient’s own immune cells and let the body build the therapy. In this review, we compare the leading delivery platforms—retargeted lentiviral vectors, engineered AAVs, LNPs (mRNA/circRNA), polymeric nanoparticles, and bioinspired systems—through the lenses of specificity, expression durability, redosing, manufacturability, immunogenicity, safety, and clinical maturity. Early human observations in hematologic malignancies and autoimmune disease show what’s possible; solid-tumor progress is still largely preclinical. Key translational challenges include: off-target transduction and murky biodistribution, liver-skewed exposure for non-viral systems, integration risk for durable vectors, repeat-dose immunity, CMC consistency, and regulatory fit. We outline a practical roadmap—quantitative biodistribution and potency, cargo-tuned persistence, receptor-aware targeting, robust CMC, and indication-specific risk–benefit—to support reproducible and clinically interpretable therapeutic development.

## 1 Introduction

CAR-T cell therapy has changed the treatment landscape for selected hematologic malignancies, especially relapsed or refractory B-cell acute lymphoblastic leukemia, diffuse large B-cell lymphoma, and multiple myeloma (1, 2). Its success has also exposed a practical limitation of the current model. Most approved CAR-T products are manufactured ex vivo: patient cells are collected, genetically modified, expanded, tested, and reinfused. This process can take weeks, requires specialized facilities, and remains difficult to scale across centers and health systems (3–6). For patients with rapidly progressive disease or poor T-cell fitness, these constraints can directly limit access to treatment.

*In vivo* CAR-T engineering was developed to address this bottleneck. The central idea is simple: instead of manufacturing CAR-T cells outside the body, a vector delivers CAR-encoding material to immune cells inside the patient (6, 7). If successful, this strategy could transform CAR-T therapy from a bespoke cell-manufacturing procedure into a programmable delivery platform. It could also support repeat dosing, lower production complexity, and faster treatment initiation (8–12).

The field is not defined by one technology. Retargeted lentiviral vectors offer durable expression but raise questions about genomic integration, redosing, and manufacturing control. LNPs provide non-integrating and potentially redosable RNA delivery, but expression is transient and biodistribution remains imperfect. Engineered AAVs, polymeric nanoparticles, extracellular-vesicle-based systems, and other bioinspired platforms add further options, each with distinct strengths and liabilities. For this reason, *in vivo* CAR-T is best understood as a set of delivery strategies rather than a single therapeutic modality.

The disease context also matters. Hematologic malignancies are currently the most advanced setting because circulating tumor cells and validated B-cell antigens are more accessible to engineered immune effectors. Solid tumors pose a different problem: delivery into dense, immunosuppressive, and spatially heterogeneous tissue remains a major barrier. Autoimmune disease introduces a third logic. Here, the goal may not be durable tumor surveillance, but time-limited immune depletion or rebalancing of pathogenic immune compartments. Whether this can produce durable immune reset remains an open clinical question. A platform that is suboptimal for long-term cancer surveillance may be well suited for reversible immune modulation.

This review provides a critical overview of *in vivo* CAR-T engineering across platforms and disease settings. We compare viral, non-viral, polymeric, and bioinspired delivery systems; summarize current progress in hematologic malignancies, solid tumors, and autoimmune diseases; and highlight the translational barriers that will determine whether early signals can become reproducible clinical benefit.

## 2 Review methodology and evidence-selection framework

This article is a narrative review, not a systematic review or meta-analysis. The aim was to synthesize the current state of *in vivo* CAR-T engineering across delivery platforms and disease contexts, with particular attention to translational maturity and evidence quality. We therefore avoid claiming systematic comprehensiveness and instead use an evidence-stratified approach to distinguish peer-reviewed experimental work from early clinical, registry-based, conference-reported, or company-disclosed information.

Literature and program searches were conducted through PubMed, Google Scholar, ClinicalTrials.gov, major oncology and immunology conference abstract sources, journal websites, and publicly available company materials. The final search update was performed on 28 June 2026. Search terms were combined around three concept groups: (1) technology terms, including “*in vivo* CAR-T”, “*in vivo* CAR T”, “CAR mRNA”, “CAR mRNA LNP”, “targeted lipid nanoparticle”, “lentiviral vector”, “AAV”, “polymeric nanoparticle”, “extracellular vesicle”, and “bioinspired vector”; (2) target or platform terms, including “CD19”, “BCMA”, “CD20”, “CD22”, “FAP”, “TRP1”, “CD3-targeted”, “CD5-targeted”, “CD7-targeted”, and “CD8-targeted”; and (3) disease terms, including “hematologic malignancy”, “multiple myeloma”, “B-cell lymphoma”, “solid tumor”, “melanoma”, “autoimmune disease”, “systemic lupus erythematosus”, “fibrosis”, and “MASH”. Program-specific searches were also performed for candidates discussed in the manuscript, including JY231, ESO-T01, KLN-1010, HN2301, CPTX2309, AERA-109, INT2104, UB-VV100/UB-VV111, and UB-VV400.

Sources were included when they directly addressed *in vivo* generation of CAR-expressing T cells, targeted delivery of CAR-encoding nucleic acids, preclinical or clinical *in vivo* CAR-T activity, or platform features relevant to *in vivo* CAR-T translation. We also included selected background literature on vector biology, LNP formulation, CAR-T safety, and regulatory or manufacturing constraints when needed to interpret the field. We excluded studies focused solely on conventional ex vivo CAR-T manufacturing, non-CAR immune engineering, or non-T-cell CAR platforms unless they directly clarified a delivery principle relevant to *in vivo* CAR-T. After scope tightening, non-T-cell CAR engineering examples such as CAR-macrophage or general myeloid reprogramming were not used as primary evidence for *in vivo* CAR-T activity. English-language sources were used for the final evidence synthesis.

Conference abstracts, preprints, company disclosures, investor presentations, press releases, and trial-registry entries were treated as non-peer-reviewed or preliminary sources. These materials were used only when they documented active clinical or translational programs, provided otherwise unavailable trial or program details, or captured rapidly evolving first-in-human activity. They were not treated as equivalent to peer-reviewed efficacy or safety evidence. Ongoing clinical programs were checked against public trial registries, sponsor materials, conference abstracts, and, where available, peer-reviewed reports. Claims based only on company or conference material were described as company-reported, conference-reported, registry-stage, or preliminary.

Evidence was classified using the following framework. Level 1 denotes peer-reviewed preclinical or mechanistic evidence. Level 2 denotes peer-reviewed or otherwise publicly documented early clinical observations with patient-level relevance but limited maturity. Level 3 denotes company-disclosed, conference-reported, registry-based, or trial-initiation information that has not yet matured into peer-reviewed clinical evidence. Level 4 denotes forward-looking hypotheses, platform concepts, or design directions that remain speculative or primarily conceptual. When a program was supported by more than one type of source, the text indicates the highest mature evidence level while retaining caveats for non-peer-reviewed claims.

## 3 Vector innovations for *in vivo* CAR-T engineering

Before discussing individual platforms, it is important to compare them using a common decision-oriented framework rather than treating each as a self-contained technology silo. For translational purposes, the most useful comparative dimensions are targeting specificity, cargo capacity, duration of CAR expression, feasibility of repeat dosing, manufacturability at scale, immunologic liabilities, dominant safety risk, and current clinical maturity. **Table 1** summarizes these variables across the major *in vivo* CAR delivery classes and should be read as the organizing scaffold for the platform discussion that follows.

**Table 1:** Comparison of lead *in vivo* CAR-T delivery platforms.

Comparative dimension	Targeted LVs (tLVVs)	Engineered AAVs	Targeted LNPs (mRNA/circRNA)	Polymeric NPs	Bioinspired vectors
Targeting specificity	Can be increased by pseudotyping and ligand retargeting, but off-target transduction remains a clinically relevant safety concern	Capsid engineering can improve tropism, yet true T-cell selectivity in vivo remains difficult	Improves with ligand engineering, but systemic liver uptake and non-specific distribution remain important constraints	Specificity depends on polymer chemistry, formulation, and surface-ligand design and remains less extensively validated in vivo	Specificity depends on carrier source, purification, and surface engineering; standardized targeting performance has not been established
Cargo capacity	Accommodates complete CAR cassettes and selected multi-component constructs within lentiviral packaging constraints	Constrained by small packaging capacity, especially for scAAV, which accelerates expression but roughly halves usable cargo capacity	Suitable for mRNA/circRNA payloads; flexible for transient constructs but limited by RNA stability and formulation efficiency	Can accommodate larger nucleic-acid payloads, depending on polymer chemistry and formulation	Variable and often limited by loading efficiency
CAR expression duration	Long-term or potentially durable because of genomic integration	Predominantly episomal and potentially longer than mRNA, but less durable in proliferating T cells than integrating systems; low integration can occur	Typically transient for mRNA; circRNA may modestly extend expression but remains non-integrating	Variable; often transient unless combined with transposon or nuclease payloads	Usually transient and strongly platform-dependent
Repeat dosing feasibility	Limited by anti-vector immunity and integration-related safety concerns	Frequently restricted by neutralizing antibodies and systemic exposure constraints	Technically feasible because the RNA cargo is non-integrating, although anti-vector or anti-ligand immunity and cumulative inflammatory toxicity may limit repeated administration	In principle redosable, but tolerability and consistency remain underdeveloped	Unclear; repeatability depends on source, immunogenicity, and manufacturing reproducibility
Manufacturing and scale-up	Requires control of targeted pseudotyping, ligand display, vector potency, residual impurities, and replication-competent vector risk	Established AAV manufacturing methods are available, but systemic dose requirements, cost, and lot potency remain barriers	Amenable to scalable manufacturing, but ligand conjugation, encapsulation consistency, particle attributes, and storage stability require lot-level control	Manufacturing can be scaled for selected formulations, but in vivo performance is supported predominantly by preclinical evidence and formulation reproducibility remains unresolved	Scale-up is limited by batch variability, purification requirements, cargo-loading variability, and incomplete potency standardization
Dominant immunologic liabilities	Anti-vector immunity; persistent mis-transduction if off-target entry occurs	Pre-existing neutralizing antibodies and immune restrictions on readministration	Innate immune activation, systemic exposure, and liver-biased distribution	Possible inflammatory or cationic-material toxicity, depending on chemistry	Source-related heterogeneity and poorly standardized immune behavior
Main safety concern	Insertional mutagenesis and long-term genomic risk	Dose-related hepatotoxicity, systemic exposure, and uncertain persistence-control balance	Hepatic accumulation, systemic exposure, and safety management across repeated dosing	Limited in vivo transduction efficiency may require higher exposure; toxicity depends on polymer chemistry and dose	Batch-dependent composition, residual biological cargo, and uncertain comparability
Best conceptual fit	Indications where durable surveillance may justify higher complexity, especially hematologic malignancy	Niche use where non-integrating delivery with tailored capsids is advantageous, while dose, redosing, and hepatic exposure remain limiting	Indications favoring transient, controllable, redosable expression, especially autoimmune disease and selected exploratory oncology uses	Preclinical optimization platform without established human in vivo CAR-T efficacy	Predominantly preclinical platform with source-dependent biological properties and unresolved manufacturing comparability
Clinical maturity (2026)	Early human feasibility signals, supported mainly by preliminary conference- or company-reported data in hematologic malignancies	Predominantly preclinical to early translational	Early clinical and translational signals, with the most developed human evidence currently reported in autoimmune disease; oncology evidence remains program-specific and limited	Predominantly preclinical	Predominantly preclinical to registry-stage for selected programs

AAV, adeno-associated virus; CAR, chimeric antigen receptor; circRNA, circular RNA; CMC, chemistry, manufacturing, and controls; LNP, lipid nanoparticle; LV, lentiviral vector; mRNA, messenger RNA; NP, nanoparticle; scAAV, self-complementary adeno-associated virus; tLVV, targeted lentiviral vector.

Viewed through this framework, targeted lentiviral vectors are strongest where durable expression and long-term surveillance are prioritized, but that advantage is inseparable from integration-related safety questions, anti-vector immunity, and manufacturing complexity. Engineered AAVs are attractive because they reduce insertional concern, yet their small cargo capacity, neutralizing-antibody constraints, and hepatic exposure profile limit how broadly they can be deployed. LNP-based systems offer the clearest case for transient and redosable *in vivo* engineering, which may be especially relevant in autoimmune disease, but their liver-biased biodistribution and limited expression duration remain major trade-offs. Polymeric systems provide high design flexibility and potentially larger cargo options, although their *in vivo* efficiency and translational maturity remain less established. Bioinspired platforms may eventually offer distinctive advantages in circulation behavior or lesion tropism, but at present they are the least standardized and the most difficult to scale reproducibly.

This comparison also helps clarify why no single platform should be presented as universally preferable. A platform that appears advantageous for hematologic malignancy may be poorly matched to autoimmune disease, and a system that is mechanistically elegant in preclinical solid-tumor models may still be impractical when judged against repeat dosing, regulatory control, or CMC consistency. Accordingly, the sections below retain platform-by-platform discussion, but each platform is interpreted through the same comparative lens summarized in **Table 1**.

A concise comparison of the major platform-level trade-offs is helpful before turning to individual systems. In general, targeted lentiviral vectors score highest on expression durability and current clinical maturity, but they do so at the cost of integration-related safety concerns, limited redosing flexibility, and more demanding manufacturing. Engineered AAVs reduce insertional concern and can show good tropism after capsid redesign, yet remain constrained by small cargo capacity, neutralizing-antibody barriers, and systemic exposure concerns, especially in the liver. LNP-based systems are strongest in redosability and non-integrating delivery, but their pharmacology is still shaped by transient expression and imperfect tissue distribution. Polymeric systems expand chemical flexibility and cargo-design options, but their clinical maturity and *in vivo* consistency lag behind the lead viral and LNP platforms. Bioinspired systems are conceptually appealing because of their membrane biology and potential lesion tropism, yet they currently face the greatest challenges in batch standardization, manufacturing reproducibility, and translational comparability.

**Figure 1:**
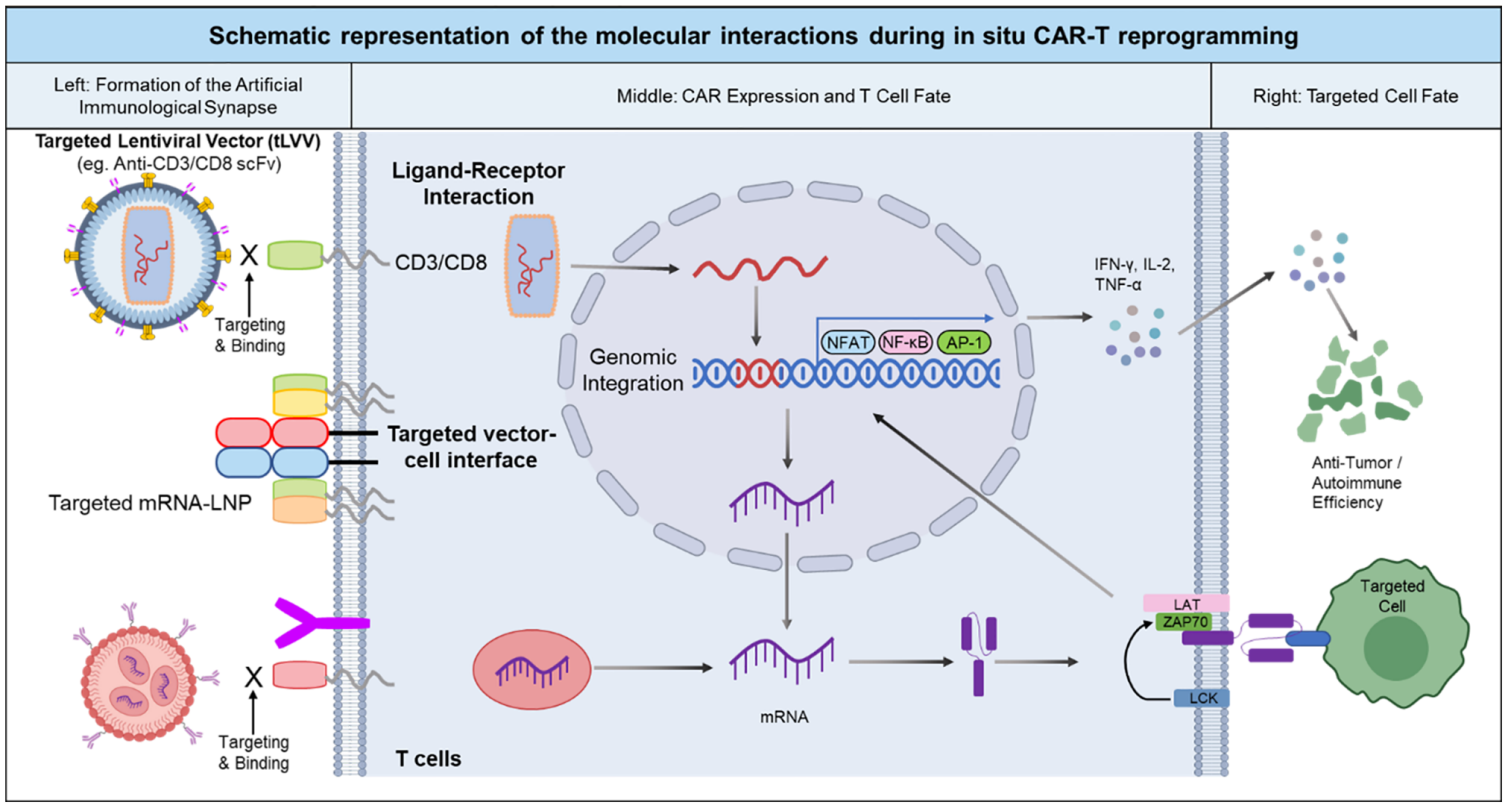
Targeted vector-cell interface during *in vivo* CAR-T engineering. The schematic illustrates ligand-mediated attachment, cellular uptake, intracellular processing, and CAR expression after *in situ* delivery. (Left) Targeted lentiviral vectors (tLVVs) and mRNA-LNPs engage host T cells through surface-conjugated ligands such as anti-CD3 or anti-CD8 scFv. This interaction is a targeted vector-cell interface, not a physiological immunological synapse. (Middle) Integrating viral payloads can support durable CAR persistence through genomic insertion, whereas mRNA delivery induces a transient expression pulse with greater reversibility. Nuclear signaling elements such as NFAT, NF-κB, and AP-1 represent downstream CAR/T-cell activation programs and should not be interpreted as mechanisms of genomic integration. (Right) Therapeutic activity depends on productive CAR expression, target engagement, and controlled T-cell activation in the relevant disease context. Created by the authors using Microsoft PowerPoint; no previously published material was reproduced or adapted. tLVV, targeted lentiviral vector; LNP, lipid nanoparticle; scFv, single-chain variable fragment; CAR, chimeric antigen receptor; NFAT, nuclear factor of activated T cells; NF-κB, nuclear factor kappa-light-chain-enhancer of activated B cells; AP-1, activator protein 1. Made by PowerPoint software; no previously published material was reproduced or adapted.

For that reason, the reader should be able to move back and forth between **Table 1** and the narrative discussion with the same set of questions in mind: How selective is the platform *in vivo*? How much cargo can it realistically carry? How long is CAR expression likely to last? Can it be redosed? How difficult is it to manufacture reproducibly? What is its dominant immunologic or safety liability? And how close is it to credible clinical deployment? The platform-specific sections below are therefore written not only to describe mechanism, but also to answer those comparative questions. As illustrated in **Figure 1**, the core platform logic discussed in this section centers on how targeted delivery, intracellular processing, and expression kinetics jointly determine whether *in vivo* engineering yields durable genomic persistence or a transient but more controllable therapeutic pulse.

### 3.1 Delivery receptor versus CAR antigen

A central distinction in *in vivo* CAR-T engineering is that the receptor used to deliver the vector is not the same as the antigen recognized by the expressed CAR. Delivery receptors such as CD3, CD5, CD7, or CD8 are used to direct a viral vector, LNP, or other carrier into an endogenous immune-cell population. CAR antigens such as CD19, CD20, BCMA, CD22, FAP, or TRP1 are the disease-associated targets recognized only after CAR expression. Conflating these two layers can obscure both safety and mechanism: a CD8-targeted LNP carrying an anti-CD19 CAR mRNA is a CD8-cell delivery system, not a CD8-directed CAR.

The delivery receptor is not biologically neutral. CD3 targeting can promote efficient T-cell entry because it engages the TCR-CD3 complex, but this same biology can trigger receptor clustering, internalization, TCR signaling, and activation-state changes. Anti-CD3 engagement has been used clinically and experimentally to modulate T-cell responses. Depending on antibody format, mode of presentation, Fc-receptor engagement, and the presence or absence of co-stimulatory signals, CD3 targeting can promote T-cell activation, generate weaker or abortive signaling, or contribute to tolerance-associated outcomes, such as anergy, apoptosis, and regulatory T-cell expansion (13). For *in vivo* CAR-T delivery, this means that CD3-targeted particles may alter the activation threshold, tonic signaling state, and early exhaustion risk of the very cells being engineered. The density, valency, and orientation of CD3-binding ligands on a vector surface are therefore not only delivery parameters; they are also pharmacologic variables that may shape vector uptake and T-cell function.

CD5 targeting raises a different issue. CD5 is broadly expressed on T cells and acts as a regulator of TCR signaling and T-cell development; its expression reflects TCR signal strength and participates in tuning immune activation (14). Because CD5 is also found on thymocytes and some B-cell compartments, CD5-directed delivery can expand the accessible cell pool but cannot be assumed to be T-cell-exclusive. Engagement of CD5 may also affect basal signaling, survival, and differentiation state, which is particularly relevant for repeated or high-density ligand exposure (15). Thus, CD5 targeting should be described as a strategy for broad T-cell-biased delivery with potential developmental and subset spillover, not as a purely inert homing tag.

CD7 targeting is even broader. CD7 is expressed on normal T cells, NK cells, thymocytes, and progenitor populations, and CD7-directed CAR-T strategies have required special attention to fratricide and lineage cross-reactivity in other settings (16). In the context of *in vivo* engineering, CD7-targeted delivery may intentionally generate a mixed T/NK effector product, as seen in CD7-targeted lentiviral approaches, but this also complicates interpretation of pharmacology and safety (17). The engineered product is no longer a uniform CAR-T population; it may include CAR-T and CAR-NK components with different expansion kinetics, cytotoxic programs, cytokine profiles, persistence, and tissue-homing behavior.

CD8 targeting enriches cytotoxic T-cell programming and is attractive for antitumor activity, but it also imposes a subset bias. CD8+ T cells are heterogeneous, including naive, effector, memory, exhausted, tissue-resident, and regulatory-like subsets with different proliferative capacity, cytokine programs, and exhaustion susceptibility (18). A high percentage of CD8-biased delivery in mouse or humanized-mouse models therefore does not prove that the clinically engineered product will be uniformly cytotoxic or durable. It indicates enrichment of a receptor- defined population whose functional state still depends on disease context, prior antigen exposure, inflammatory signals, and the delivered CAR payload.

For these reasons, selective transduction should not be equated with exclusive transduction. Percentages from mouse, humanized-mouse, or NHP studies are useful measures of model-specific delivery bias, but they should not be treated as proof of clinical specificity in humans. Off-target or unintended engineering of regulatory T cells, NK cells, thymocytes, progenitor-like populations, or other receptor-positive cells remains a program-specific risk that must be evaluated empirically for each carrier, ligand, dose, route, and disease context.

### 3.2 Viral vectors: key advances and optimization

#### 3.2.1 Lentivirus

When judged against the comparative dimensions in **Table 1**, lentiviral vectors are defined by three linked features: relatively high cargo flexibility, the potential for long-duration CAR expression through genomic integration, and the earliest meaningful movement toward human clinical use. Those same features also explain their main liabilities, namely integration-related safety concerns, limited redosing flexibility, and manufacturing complexity.

Lentivirus (LV) has emerged as the most mature and widely investigated viral vector for *in vivo* CAR-T engineering, owing to its ability to stably integrate the CAR transgene into the genome of both dividing and non-dividing T cells, enabling long-term persistence of functional CAR-T cells, a persistence-oriented logic summarized in **Figure 1** (10, 19, 20). A central breakthrough in LV-based *in vivo* CAR-T lies in the engineering of viral envelope glycoproteins, which determines cell tropism and transduction efficiency—addressing the inherent broad tropism of wild-type LVs that limits T cell-specific targeting (10). The most extensively optimized envelope protein is vesicular stomatitis virus glycoprotein (VSV-G), whose native binding to low-density lipoprotein receptor (LDLR) (expressed on most somatic cells) leads to severe off-target transduction. To overcome this, site-directed mutagenesis of key residues (K47, R354) in VSV-G has been developed to abrogate LDLR binding while preserving its fusogenic function, laying the foundation for T cell-specific retargeting (17, 21–23).

To achieve precise T cell targeting, modified VSV-G-enveloped LVs are further engineered to display high-affinity ligands on their surface, including single-chain variable fragments (scFvs), designed ankyrin repeat proteins (DARPins), or variable domains of heavy-chain-only antibodies (VHHs) that recognize T cell-specific markers (24–26). CD3, as a pan-T cell receptor complex, is a common targeting moiety—LVs displaying anti-CD3 scFv have been shown to bias delivery toward CD3^+^ T cells *in vivo (*27). CD8-targeted LVs, using CD8-specific scFv or DARPins, have achieved high CD8-biased delivery in humanized mouse models (28). Additionally, CD7-targeted LVs (e.g., Interius BioTherapeutics’ INT2104) have been developed to co-transduce T cells and natural killer (NK) cells, expanding the spectrum of effector cells for anti-tumor immunity while also creating a mixed engineered-cell product (17).

Beyond VSV-G, envelope glycoproteins from paramyxoviruses (Nipah virus (NiV) and Measles virus (MV)) have gained attention for LV pseudotyping, as their functionally segregated attachment (G protein) and fusion (F protein) glycoproteins simplify engineering. This modular separation allows natural receptor usage to be ablated in the attachment protein and replaced with a high-affinity targeting domain while preserving fusion activity (28, 29). NiV-G protein, when mutated to abrogate binding to its native receptors (ephrin-B2/B3), can be engineered to display T cell-specific binders, while NiV-F protein mediates membrane fusion. This separation enables retargeted LV entry into selected cell populations, as shown by CD8-targeted NiV-LVs (30–32). MV-H, engineered to ablate natural receptor recognition and fused to a CD4-specific DARPin, has been used to generate CD4-targeted LVs. These vectors selectively transduced human CD4+ T cells at approximately 55% efficiency in stimulated peripheral blood mononuclear cells (PBMCs) *in vitro* and achieved *in vivo* transduction of human CD4+ cells in humanized mouse models, reaching up to 16% in stimulated PBMC-engrafted mice (33–35). These paramyxovirus-enveloped LVs exhibit reduced serum inactivation and lower pre-existing neutralizing antibody levels in humans, enhancing their *in vivo* persistence (31).

Optimization of the LV payload design further enhances *in vivo* CAR-T performance. In DNA-based or integrating vector systems, T cell-specific promoters can restrict transcriptional CAR expression after nuclear delivery or genomic integration, thereby reducing ectopic expression in non-target cells (36, 37). This promoter logic applies to DNA-based constructs and should not be generalized to directly delivered mature CAR mRNA, where cell selectivity is determined primarily before or during delivery rather than at the transcriptional promoter level. Additionally, incorporating co-stimulatory domains (4-1BB or CD28) into the CAR construct, along with optimized hinge and transmembrane domains, improves CAR-T cell expansion, persistence, and anti-tumor efficacy (38). Nicolai et al. developed a lentiviral platform, for instance, that includes CD80 and CD58 co-stimulatory ligands in the LV envelope, providing dual activation signals to T cells and enhancing *in vivo* CAR-T generation (3). To mitigate macrophage-mediated clearance of LVs, incorporating the “don’t eat me” signal CD47 into the viral envelope has been shown to reduce vector uptake by phagocytes, improving LV bioavailability *in vivo (*39).

Safety enhancements are critical for LV-based *in vivo* CAR-T translation. Self-inactivating (SIN) LVs, with deletions in the long terminal repeat (LTR), reduce LTR-associated enhancer and promoter activity and lower vector-related transcriptional activation risk, but they do not eliminate insertional mutagenesis or integration-related clonal risk (40, 41). When LV delivery is combined with CRISPR-Cas9-mediated targeted integration, the site-specific genomic insertion is driven by the editing system and donor design rather than by an intrinsic non-random behavior of the carrier itself (42).

Available preclinical data support the feasibility of engineered LVs for *in vivo* CAR-T generation, whereas the clinical evidence remains early and limited in scale. Using the VivoVec platform, a third-generation self-inactivating LV displaying an anti-CD3 scFv together with CD80/CD58 costimulatory domains and pseudotyped with cocal fusion glycoprotein, Nicolai et al. generated anti-CD20 CAR T cells in nonhuman primates without lymphodepleting chemotherapy. In NHPs, VivoVec particles incorporating a multidomain fusion ligand generated CAR+ cells comprising up to 65% of circulating T cells and produced complete B-cell depletion for up to 76 days (3). Clinically, Interius’ INT2104 (CD7-targeted LV delivering anti-CD20 CAR) initiated phase I trials in 2024 for relapsed/refractory B cell malignancies, with preclinical data showing specific T/NK cell transduction and B cell depletion (17). Umoja’s UB-VV111 (anti-CD3 scFv LV delivering anti-CD19 CAR and rapamycin-activated cytokine receptor (RACR)) entered phase I in 2024. Publicly available support for this program is still weighted toward preclinical models and development-stage disclosures rather than mature peer-reviewed human efficacy data.

#### 3.2.2 Adeno-associated virus

Relative to lentiviral systems, AAV occupies a different position in the comparison framework: it is attractive because it reduces insertional risk and can be retargeted through capsid engineering, but it is more limited in cargo capacity, is harder to redose in the presence of neutralizing antibodies, and remains constrained by systemic exposure and hepatic liability.

Adeno-associated virus (AAV) is an important predominantly episomal viral platform under investigation for *in vivo* CAR-T engineering. Its appeal lies in reduced insertional concern relative to lentiviral systems, but recombinant AAV should not be described as absolutely non-integrating because rare integration events and integration at DNA break sites can occur. Its practical value is further tempered by cargo-size constraints, systemic exposure issues, and still-limited clinical translation (43). Unlike wild-type AAV, which exhibits broad tissue tropism but poor T cell transduction efficiency, targeted AAV engineering focuses on capsid modification to achieve T cell-specific delivery, a key prerequisite for clinical translation.

A central strategy for AAV tropism optimization is site-directed mutagenesis of capsid proteins (VP1/VP2/VP3) to abrogate binding to native receptors, eliminating off-target transduction. For example, mutating residues R585A/R588A in AAV2 capsid disrupts heparan sulfate binding, while V473D/K531E mutations in AAV6 abrogate sialic acid and heparin binding—these modifications lay the foundation for T cell-specific retargeting (44–46). To further enhance T cell selectivity, modified AAV capsids are engineered to display T cell-specific ligands, which are inserted into the surface-exposed GH2/GH3 loop of VP1, a conserved region that preserves capsid core integrity and unimpaired gene delivery activity (47). CD8-targeted AAVs, for instance, incorporate CD8-specific designed ankyrin repeat proteins (DARPins) into the GH2/GH3 loop, achieving >80% CD8-biased transduction in immunocompetent mouse models (47). Beyond CD8, other T-cell-associated markers have also been explored to broaden T-cell delivery. Anti-CD3-directed delivery platforms, including EDV/AAV two-vector systems, have enabled *in vivo* engineering of both CD4+ and CD8+ T cells, whereas CD4-specific nanobody-modified AAV2 vectors have improved selective transduction of human CD4+ T cells *in vitro (*42, 48). Additionally, capsid evolution through directed evolution or structure-guided design has produced AAV variants with apparent T cell tropism in mouse systems. AAV-Ark313, identified through VP3 saturation mutagenesis, efficiently transduced murine T cells without exogenous ligands and, in its original experimental context, supported T cell receptor alpha constant (TRAC) locus CAR integration when paired with genome-editing machinery (49).

Payload design is also important for AAV-based *in vivo* CAR-T strategies. Because AAV delivers DNA and is constrained by a limited packaging capacity of approximately 4.7 kb, donor cassettes often require compact design, including minimized regulatory elements, compact CAR architectures, and shortened homology arms where appropriate. In site-specific approaches, AAV can serve as the donor-template vector, whereas CAR integration into loci such as TRAC is driven by the accompanying nuclease/editing system and homology-directed repair rather than by the episomal biology of AAV itself. Promoterless TRAC-targeted donor designs can further restrict CAR expression to correctly edited T cells (42, 49).

Safety enhancements for AAV include reducing pre-existing neutralizing antibody (NAb) interference—capsid glycosylation or PEGylation masks antigenic epitopes, improving vector bioavailability in patients with high NAb titers (50–53). Additionally, self-complementary AAV (scAAV) vectors form double-stranded DNA without host cell DNA synthesis and can accelerate transgene expression, but this advantage comes at the cost of approximately halving the already limited AAV packaging capacity. This substantially constrains delivery of full CAR cassettes and makes scAAV better suited to compact regulatory or editing components than to large multi-domain CAR payloads (54, 55).

Current evidence for AAV in this field remains predominantly preclinical. Targeted AAV variants outperform unmodified parental serotypes in selective gene-transfer experiments (47), and mouse studies support the feasibility of *in vivo* T-cell engineering, antitumor activity, and B-cell depletion with improvement of lupus-associated pathology in selected models (56–58). In an early proof-of-concept study, systemic AAV delivery of a CD4-CAR into humanized NCG-HuPBL mice bearing human T-cell leukemia generated CAR-expressing immune cells *in vivo*, depleted CD3+CD4+ leukemic targets, reduced whole-body tumor bioluminescence and tumor burden across organs, and significantly improved survival compared with untreated controls (56). More recently, AAV6-M2-mediated *in vivo* CD19-CAR generation in humanized immune-system mice produced robust B-cell depletion; in HIS-SLE(Systemic Lupus Erythematosus) mice, AAV6-M2-CAR depleted circulating and tissue-resident B cells and ameliorated lupus-associated pathology (57). In an EDV/AAV TRAC-CAR platform, preclinical nonhuman-primate data further reported complete peripheral CD20+ B-cell aplasia by day 10 after a single intravenous dose, although these results remain company-reported and require peer-reviewed validation (42).

### 3.3 Non-viral vectors: mainstream platforms and breakthroughs

#### 3.3.1 Lipid nanoparticles: targeted delivery and mRNA/circRNA cargo optimization

In the comparative framework, LNPs are the clearest example of a platform whose main strengths lie in non-integration, repeat dosing, and relative manufacturing tractability rather than in durable expression. Their trade-off is that expression is usually transient and tissue exposure remains imperfect, with liver-biased distribution still shaping real-world performance.

Lipid nanoparticles (LNPs) currently represent one of the most active non-viral development paths for *in vivo* CAR engineering because they are non-integrating, comparatively manufacturable, and in principle amenable to repeat dosing. At the same time, their practical advantages must be weighed against persistent liver-biased distribution, transient expression kinetics, and formulation-sensitive performance. This more transient engineering logic is also depicted in **Figure 1**. The core composition of therapeutic LNPs typically includes ionizable lipids, phospholipids, cholesterol, and PEG-lipids, with structural optimization focused on enhancing T cell targeting specificity and nucleic acid stability (59).

Targeted delivery of LNPs to T cells is primarily achieved through surface modification with T cell-specific ligands, among which CD3, CD4, CD5, CD7 and CD8 ligands are the most widely explored due to their restricted expression on T cell subsets and functional relevance (60–64). CD8-targeted LNPs, often conjugated with anti-CD8 antibodies, single-chain variable fragments (scFv), or variable domains of heavy-chain-only antibodies (VHHs), enable selective delivery to CD8^+^ T cells—critical for cytotoxic anti-tumor immunity. For example, In Capstan’s CD8-targeted L829 tLNP platform, anti-CD8 antibody-conjugated LNPs delivered anti-CD19 CAR mRNA preferentially to CD8+ T cells. In PBMC-humanized mice, a single 10–30 µg dose induced rapid near-complete B-cell depletion within hours, with CAR expression peaking at approximately 6 h and remaining detectable at 24 h. In non-human primate (cynomolgus monkey) studies, a 3-dose regimen induced profound peripheral B-cell depletion within 24 hours, with B-cell reconstitution initiating around day 21 and returning to near-baseline levels by day 35. No central nervous system toxicity was observed; however, one animal in the 1.5 mg/kg dose group developed severe immune effector cell-associated hemophagocytic lymphohistiocytosis (HLH)-like syndrome, while all other animals exhibited only mild, transient elevations in cytokines and liver enzymes (9). Because mature CAR mRNA is translated directly in the cytoplasm, T-cell specificity is determined mainly by particle tropism, ligand-mediated uptake and intracellular delivery, rather than by T-cell-specific promoters used in DNA-based constructs (9). Post-entry expression can be further modulated by untranslated regions, codon optimization, RNA chemistry, and microRNA-responsive elements, but these mechanisms are distinct from promoter-mediated transcriptional restriction (65).

CD5-targeted LNPs, leveraging CD5’s pan-T cell expression (and limited expression on B cells and thymocytes), have shown utility in broader T cell engineering scenarios, including autoimmune diseases and fibrosis (9, 66). For instance, CD5-targeted LNPs delivering platelet-derived growth factor receptor β (PDGFRβ)-CAR mRNA directly reprogrammed T cells *in vivo* to eliminate ECM-producing cells (e.g., fibroblasts, pericytes), reversing multi-organ fibrosis (including cardiac fibrosis) in mouse models of chronic kidney disease (66). Ligand format is an important design variable for T-cell-targeted LNPs. CD5-targeted LNPs using antibody-derived targeting moieties have demonstrated efficient T-cell mRNA delivery and *in vivo* CAR-T generation in preclinical models. Such smaller formats such as scFv or VHH may offer potential advantages in particle decoration, steric accessibility, manufacturability and aggregation control (62, 63, 67).

Cargo optimization is a critical determinant of LNP-based *in vivo* CAR-T performance, with mRNA and circular RNA (circRNA) being the primary payloads due to their cytoplasmic expression (avoiding nuclear entry) and safety profile (10). mRNA cargo is typically modified with synthetic nucleotides (e.g., pseudouridine, 5-methoxyuridine) to reduce recognition by innate immune sensors (TLR3/7/8), minimizing immunogenicity while enhancing translational efficiency (68, 69). Emerging RNA formats, including circular RNA and self-amplifying RNA(saRNA), are being explored to prolong CAR expression or reduce dose requirements. CircRNA is being investigated to extend expression duration within non-integrating RNA platforms because its covalently closed structure resists exonuclease degradation (70). Available preclinical comparisons with linear mRNA suggest longer-lasting CAR expression and improved functional persistence in model systems. In a NALM-6 leukemia xenograft model, circular mRNA-derived anti-CD19 CAR-T cells showed lower tumor burden, higher CAR-T persistence, increased memory T-cell proportions, and prolonged survival compared with linear mRNA CAR-T cells (70). A separate tLNP-circCAR platform also reported complete splenic B-cell depletion and complete remission of CD19+ NALM-6-Luc tumors in humanized mice after a single intravenous dose, although these data are currently available as a conference abstract (71).

LNP-based *in vivo* CAR engineering now has both substantial preclinical support and early translational or clinical signals, but the strength of evidence differs markedly by program. MagicRNA’s HN2301 (CD8-targeted LNP delivering anti-CD19 CAR mRNA) provides a peer-reviewed early human signal for autoimmune application in refractory SLE, but the reported cohort remains very small and follow-up remains short (72). Capstan’s CPTX2309 (CD8-targeted LNP-CD19 CAR) is important as a development-stage program supported by ACR preclinical/translational abstracts and a phase 1 initiation disclosure, although currently available information should not be treated as established clinical efficacy (73).

#### 3.3.2 Polymeric nanoparticles (PβAEs, PEI): biodegradability and low immunogenicity

Polymeric nanoparticles offer a chemically flexible and potentially complementary alternative to LNPs for *in vivo* CAR engineering. Their key appeal is design modularity, especially for larger cargos or delivery architectures that are difficult to implement with standard LNPs, but most evidence remains preclinical and their *in vivo* performance is still less mature than that of leading LNP systems (74). Among mainstream polymeric materials, poly(β-amino esters) (PβAEs) and polyethyleneimine (PEI) are the most extensively explored for T cell engineering, with structural optimization focused on reducing cytotoxicity, enhancing T cell targeting, and improving endosomal escape.

Poly(β-amino ester) (PβAE) nanoparticles are attractive for *in vivo* T-cell reprogramming because their protonatable tertiary amines facilitate endosomal escape, whereas biodegradable ester linkages reduce long-term polymer persistence. In preclinical studies, PβAE-based nanoparticles functionalized with antibody-derived T-cell targeting moieties, such as anti-CD3 or anti-CD8 antibodies, have delivered CAR- or TCR-encoding mRNA to circulating T cells and achieved transient *in vivo* T-cell reprogramming. PβAE DNA nanoparticles have also been explored with transposon-based CAR systems, but these studies remain preclinical feasibility data, with efficiency, toxicity, reproducibility and scale-up still unresolved (75).

Polyethyleneimine (PEI), a classic cationic polymer with high amine density, is widely used for *in vivo* gene delivery via chemical modification to mitigate inherent cytotoxicity. In the context of *in vivo* CAR-T, relevant optimization strategies include PEGylation to reduce non-specific protein adsorption and conjugation with T cell ligands (e.g., anti-CD3 scFv) to improve T-cell targeting. PEI-based nanoparticles have also been combined with mRNA cargo. Star-shaped multi-arm polyaspartamide nanoparticles delivering CD19 CAR minicircle DNA (mcDNA) achieved substantial and potentially durable CAR expression in immune cells without requiring viral integration machinery, and supported repeated dosing in mice, with efficacy still under evaluation in syngeneic autoimmune disease models (76).

Cargo optimization for polymeric nanoparticles focuses on plasmid DNA (pDNA), mRNA, and transposon systems, with tailored designs to match polymer properties. PβAEs exhibit high pDNA condensation efficiency, making them suitable for large cargo (e.g., CAR transposon + transposase, ~10 kb), while PEI’s strong cationic charge enables stable mRNA complexation, protecting against enzymatic degradation (74, 77). When transposon or nuclease systems are used, any resulting genomic integration should be attributed to the transposase or genome-editing payload, not to the intrinsic behavior of the polymeric carrier. Additionally, polymeric nanoparticles have been combined with CRISPR-Cas9 tools. PBAE mRNA nanocarriers can mediate efficient TRAC locus knockout in T cells via transient delivery of megaTAL-encoding mRNA, and reprogram CAR-T cells to a central memory phenotype by delivering Foxo1_3_A mRNA. This phenotypic reprogramming markedly improves *in vivo* persistence and antitumor efficacy relative to conventionally manufactured CAR-T cells (78).

Despite advantages, polymeric nanoparticles face challenges including inherent cytotoxicity, especially high-molecular-weight PEI, and lower *in vivo* transduction efficiency than LNPs (74, 79). Optimization strategies for PEI-based nanoparticles include tuning the molecular weight within the 5–25 kDa range to balance transfection efficiency and cytotoxicity, using low-molecular-weight PEI (800 Da) combined with low-generation PAMAM dendrimers to reduce cytotoxicity while maintaining gene delivery efficacy, incorporating hydrophilic PEG segments to construct amphiphilic copolymers for micellar encapsulation to mitigate cationic charge-related toxicity, and optimizing polymer topological structure (using branched/non-linear PEI rather than linear PEI of the same molecular weight) to improve transfection efficiency and reduce cytotoxicity (80–83).

#### 3.3.3 Bioinspired vectors (EVs, erythrocyte-mimetic NPs)

In comparative terms, bioinspired vectors are currently the most conceptually distinctive but also the least standardized class in **Table 1**. They may eventually offer advantages in circulation behavior, immune compatibility, or lesion tropism, yet their current limitations in large-scale production, batch definition, and potency control are more severe than for the more established platform classes.

Bioinspired delivery vehicles are designed by borrowing structural or functional features from natural biological systems such as cell membranes and extracellular vesicles (EVs). Their appeal lies in the possibility of improved circulation behavior, immune compatibility, or lesion tropism. However, these proposed advantages remain highly context-dependent, and the class is still constrained by complex preparation, high batch-to-batch variability, uncertain cargo loading, and unresolved scale-up problems.

Synthetic PBAE-based DNA nanocarriers have been used to program T cells *in situ* by delivering plasmids encoding a leukemia-specific CAR and a hyperactive piggyBac transposase, with anti-CD3e f(ab’)_2_ fragments used for T-cell targeting (77). This example supports the feasibility of polymeric DNA delivery for *in vivo* CAR-T generation. The Erythrocyte-inspired delivery system harnesses the innate 120-day long-circulating capability and spleen homing property of red blood cells. For the purposes of this review, the relevant point is whether such biomimetic systems can support T-cell targeted CAR delivery rather than broader non-T-cell engineering. The biomimetic enveloped adeno-associated virus (AEV) vector coated with erythrocyte membrane can evade antibody-mediated neutralization and immune cell-mediated phagocytosis, and has been reported to achieve *in vivo* CAR-T generation in mouse models of B-cell lymphoma after modification with anti-CD3 antibodies (84–87). Engineered virus-like particles (eVLPs) are non-replicating particles that preserve viral particle architecture and can transiently deliver genome-editing proteins or RNPs without encoding a replicating viral genome. In principle, eVLP-delivered editors could be combined with separate donor templates to support site-specific CAR insertion, but direct evidence for efficient *in vivo* CAR-T generation using VLPs remains limited. Thus, VLPs are best viewed as an emerging editor-delivery platform rather than a validated standalone CAR delivery system (88, 89).

Small extracellular vesicles (small EVs) are frequently discussed as endogenous non-viral delivery options for *in vivo* CAR engineering. When endosomal biogenesis is demonstrated, the term exosome may be appropriate; when the biogenesis route is not established, EV or small EV is the more accurate terminology. These vesicles can display natural biocompatibility, and surface molecules such as CD47 may reduce phagocytic clearance in selected systems. However, broad claims about blood-brain-barrier crossing, lesion tropism, or immune evasion should be treated as source-, cargo-, and manufacturing-dependent rather than as class-wide properties (90, 91). Engineered EVs can encapsulate nucleic acid cargos and protect them from nuclease degradation, enabling delivery to selected host immune cells in preclinical models. However, direct evidence for EV-mediated *in vivo* CAR-T generation remains limited; current CAR-encoding EV studies more clearly support CAR mRNA delivery to myeloid/macrophage compartments rather than robust T-cell reprogramming (92). Nevertheless, their clinical translation faces core bottlenecks: low large-scale production yield, poor and variable cargo loading efficiency, insufficient *in vivo* stability, heterogeneous surface composition, and incomplete potency standardization (91). Different EV sources also create distinct safety risks, residual-cargo profiles, and characterization requirements that should follow current EV nomenclature and reporting standards (93). Further work is needed not only on engineering optimization but also on process definition, potency testing, identity characterization, and standardization before these systems can be judged on realistic translational grounds.

The comparative features and clinical status of these leading *in vivo* delivery platforms are summarized in **Table 1**.

## 4 Evidence-stratified clinical and translational landscape

Because clinical and translational programs in this field differ widely in source quality, platform maturity, and public data availability, we summarize the current landscape in an evidence-stratified table (**Table 2**). The purpose of this table is not to imply that all listed programs have established clinical efficacy. Rather, it separates peer-reviewed human evidence, official conference or company updates, registry-stage programs, and preclinical programs. Non-T-cell CAR platforms, including CAR-macrophage or myeloid-reprogramming programs, are not included as primary clinical-landscape entries because they fall outside the core scope of *in vivo* CAR-T.

**Table 2:** Evidence-stratified clinical and translational landscape of *in vivo* CAR-T programs.

Program	JY231	ESO-T01	KLN-1010	INT2104	HN2301	CPTX2309	UB-VV400
Company	Genocury	EsoBiotec	Kelonia	Interius/Kite-Gilead	MagicRNA	Capstan/AbbVie	Umoja/Abbvie
Delivery platform	CD3-targeted lentiviral vector	Nanobody-directed lentiviral vector	CD3-targeted lentiviral vector with modified VSV-G	CD7-targeted lentiviral vector	CD8-targeted LNP	CD8-targeted LNP	Surface-engineered lentiviral vector with rapamycin-activated cytokine receptor payload
Targeted endogenous cell population	T cells	T cells	T cells via CD3 targeting	CD7+ T and NK cells	CD8+ T cells	CD8+ T cells	T cells
CAR antigen	CD19	BCMA	BCMA	CD20 CAR	CD19	CD19	CD22
Disease indication	R/R B-cell lymphoma/leukemia	R/R multiple myeloma	R/R multiple myeloma	R/R B-cell malignancies	SLE	RA, SLE	R/R LBCL
Administration route	IV	IV	IV	IV	IV	IV	IV with rapamycin
Lymphodepletion/pharmacologic enrichment	Lymphodepleting and non-lymphodepleting	No lymphodepleting	No lymphodepleting	No lymphodepleting	No lymphodepleting	No lymphodepleting	Pharmacologic enrichment with rapamycin; no lymphodepleting
Trial ID/phase	NCT06678282 /pre-clinical	NCT06691685 /Phase 1	NCT07075185 /phase 1	NCT06539338 /phase 1	NCT06801119 /phase 1	NCT06917742 /phase 1	NCT06743503 /phase 1
Treated/evaluable participants	Registry planned enrollment 20; a single reported DLBCL case	4 treated; 4 evaluable for MRD assessment	18 dosed; 14 had 1-month MRD data	Not disclosed	5 refractory SLE patients	Not disclosed	Not disclosed
Data cutoff /follow-up	Short follow-up	2–3 months	1–6 months	Not disclosed	3 months	Not disclosed	Not disclosed
Clinical efficacy and safety	CR in one DLBCL patient	4/4 objective responses, MRD negativity in 4/4 evaluable patients; CRS in 4/4 (3 grade 3, 1 grade 1), one grade 1 ICANS, grade >=3 AEs in all patients	100% ORR and MRD among evaluable patients; 14/14 patients with available month-1 MRD data were MRD-negative. No grade >=3 CRS; one grade 1 and one transient grade 3 ICANS event were reported.	Not disclosed	SLEDAI-2K improvement in 5/5 at Month 3; complete peripheral B-cell depletion in 3/5 with marked reduction in 2/5 (2 mg); autoantibody reduction and complement normalization in 2/5. CRS in 3/5 ICANS 0/5; grade >=3 AEs 0/5; transient lymphopenia and CRP/IL-6 elevations observed.	Not disclosed	Not disclosed
Evidence category	Company report; Level 3	Peer-reviewed human clinical evidence; Level 2	Conference/company disclosure plus registry; Level 3	Peer-reviewed preclinical	Peer-reviewed early human clinical evidence; Level 2	Conference abstract/company disclosure/registry-stage; Level 3	Preclinical abstract plus trial registry; Level 3

AE, adverse event; BCMA, B-cell maturation antigen; CAR, chimeric antigen receptor; CD, cluster of differentiation; CR, complete response; CRP, C-reactive protein; CRS, cytokine release syndrome; DLBCL, diffuse large B-cell lymphoma; ICANS, immune effector cell-associated neurotoxicity syndrome; IL-6, interleukin 6; IRR, infusion-related reaction; IV, intravenous; LBCL, large B-cell lymphoma; LNP, lipid nanoparticle; MRD, minimal residual disease; NCT, ClinicalTrials.gov identifier; NK, natural killer; RA, rheumatoid arthritis; R/R, relapsed or refractory; RRMM, relapsed or refractory multiple myeloma; SLE, systemic lupus erythematosus; SLEDAI-2K, Systemic Lupus Erythematosus Disease Activity Index 2000; VSV-G, vesicular stomatitis virus glycoprotein G.

## 5 Cross-disease translation of *in vivo* CAR-T

Cross-disease translation is often presented as a core strength of *in vivo* CAR engineering, but the rationale for doing so differs sharply by indication. Hematologic malignancies currently provide the clearest clinical proof-of-concept because target antigens such as CD19, BCMA, and CD20 are relatively well validated and are more accessible to circulating effector cells. Solid tumors remain far less mature because antigen heterogeneity, abnormal stroma, poor deep-tissue penetration, and immunosuppressive microenvironments compound the delivery problem. Autoimmune disease represents a third and conceptually distinct use case: the therapeutic objective is not continuous tumor surveillance, but controlled immune depletion or rebalancing, often through transient depletion of pathogenic B-cell compartments.

For this reason, the sections below use explicit evidence-level labels wherever program-level maturity could otherwise be misread. We define four categories: [Level 1: peer-reviewed preclinical evidence], [Level 2: early clinical observation], [Level 3: company-disclosed or conference-reported preliminary information], and [Level 4: forward-looking hypothesis or design direction]. This framework is especially important because many current discussions of *in vivo* CAR-T merge animal proof-of-concept, first-in-human signals, corporate pipeline updates, and speculative future strategy into a single narrative, which overstates the maturity of the field.

### 5.1 Hematological malignancies

Hematologic malignancies remain the most advanced disease setting for *in vivo* CAR-T translation. Even here, however, the literature contains a mixture of stronger and weaker evidence sources. The most reliable data currently come from peer-reviewed mechanistic and animal studies, followed by small early-phase clinical reports. Company-announced conference results are useful for tracking field movement, but they should not be interpreted as equivalent to mature clinical validation.

#### 5.1.1 Core targets and current evidence status

CD19 is the best-established antigenic entry point for *in vivo* CAR-T approaches in B-cell malignancies because of its broad expression across B-cell developmental stages and the precedented activity of ex vivo CD19 CAR-T therapy (94). Peer-reviewed preclinical studies support the feasibility of generating functional CD19-directed CAR effector cells *in vivo* using targeted lentiviral and LNP-based systems (9). By contrast, clinical evidence remains limited and heterogeneous. The reported Genocury’s JY231 experience in relapsed/refractory diffuse large B-cell lymphoma provides a valuable early clinical signal, but it remains a very small dataset and should be interpreted as proof-of-feasibility rather than proof of reproducible efficacy (95). Similarly, programs such as Umoja’s UB-VV100 illustrate active clinical development, yet much of the available detail still comes from preclinical studies or company-linked disclosures rather than peer-reviewed human outcomes (96).

BCMA is the leading target for *in vivo* CAR engineering in multiple myeloma, where rapid relapse after standard therapy creates strong motivation for scalable non-autologous approaches. Peer-reviewed early clinical observations such as ESO-T01 suggest that *in vivo* BCMA-directed CAR generation is feasible and may induce meaningful responses in small cohorts, but the available studies remain too limited to establish the magnitude or durability of benefit with confidence (97). Separately, KLN-1010, another BCMA-directed *in vivo* CAR-T program, has shown encouraging preliminary activity in American Society of Clinical Oncology (ASCO) 2026/company-reported phase 1 updates, including detectable CAR-T expansion without lymphodepleting chemotherapy and minimal residual disease (MRD)-negative responses among evaluable patients (98). However, these findings are important as an early clinical signal, but they remain preliminary conference/company-reported data from an ongoing phase 1 study.

CD20 serves mainly as a complementary target in this setting, especially for mitigating CD19-negative relapse and broadening B-cell coverage. Here again, the evidence base is mixed. INT2104 is relevant as a field indicator because public sources support a CD7-targeted lentiviral vector that delivers a CD20-directed CAR transgene to T and NK cells *in vivo*. This supports the feasibility of multi-lineage immune-cell engineering and B-cell depletion in preclinical models, but it should not be described as peer-reviewed clinical efficacy evidence (17).

Relapse and resistance remain central translational challenges even if initial *in vivo* CAR generation is successful. Antigen escape, insufficient CAR persistence, functional exhaustion, and variable *in vivo* cell expansion may all erode durability. Dual-target strategies such as CD19/CD22 or BCMA/CS1 illustrate a plausible way to reduce single-antigen escape, and several peer-reviewed preclinical or small clinical reports are encouraging (99–101). However, these approaches also increase construct complexity, may intensify CMC and release-testing burdens, and can complicate interpretation of which engineering feature drove efficacy. Thus, although hematologic malignancies are clearly the leading indication, even this disease area is still in the transition from proof-of-concept to reproducible platform-level translation.

### 5.2 Solid tumors

**Figure 2:**
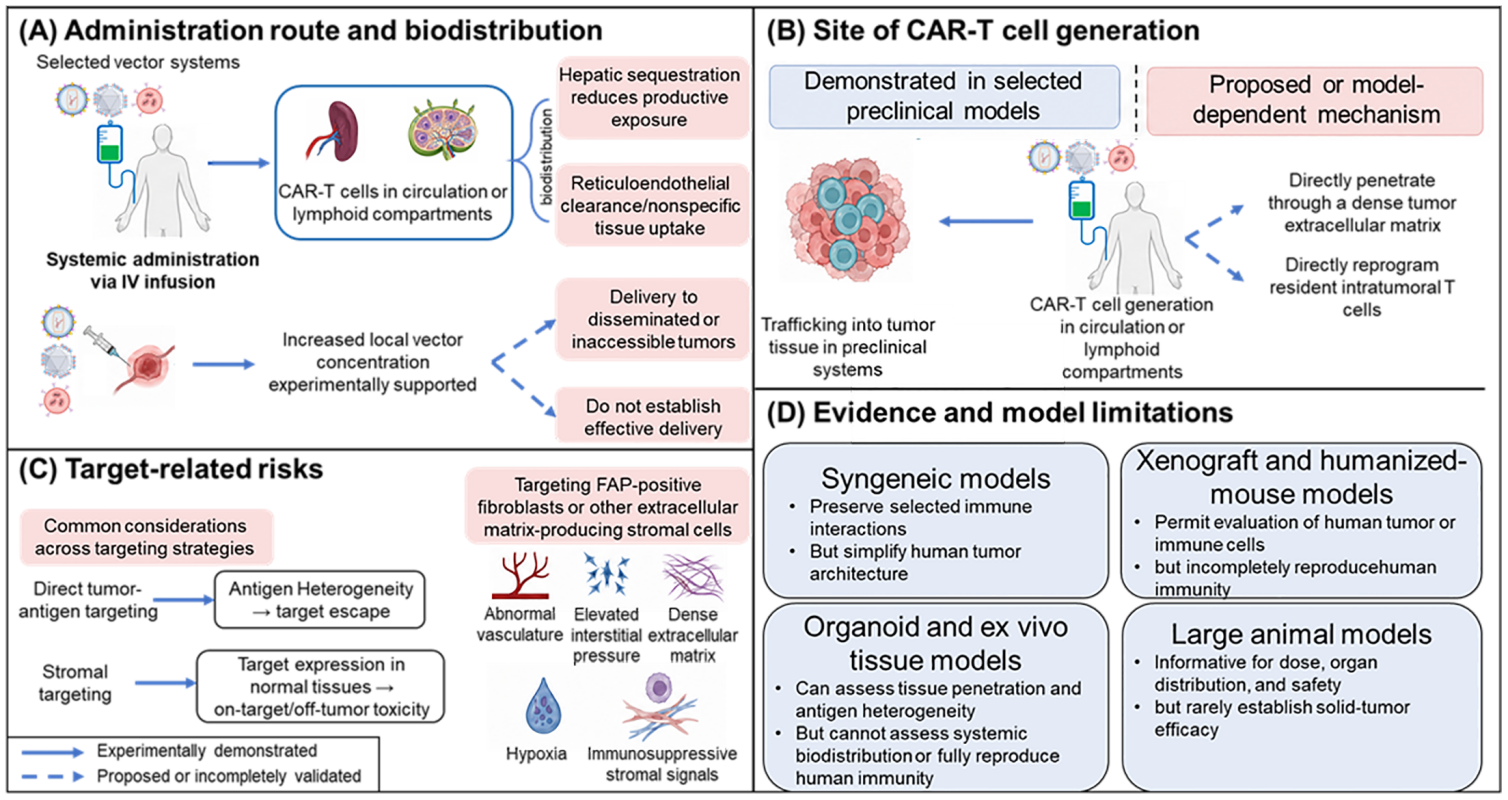
Evidence map for *in vivo* CAR-T engineering in solid tumors. **(A)** Systemic administration may generate CAR-T cells in circulating or lymphoid compartments, but hepatic sequestration and reticuloendothelial clearance can limit tumor exposure. Intratumoral or regional administration increases local exposure but may not reach disseminated or inaccessible tumors. **(B)** Circulating CAR-T generation followed by tumor trafficking has preclinical support, whereas direct penetration of dense tumor extracellular matrix and reprogramming of resident intratumoral T cells remain model-dependent. **(C)** Tumor-antigen and stromal targeting face shared risks, including target heterogeneity, escape, and on-target/off-tumor toxicity. Abnormal vasculature, interstitial pressure, dense extracellular matrix, hypoxia, and stromal signals further restrict delivery and function. **(D)** Syngeneic, xenograft or humanized-mouse, organoid or ex vivo tissue, and large-animal models address complementary aspects of immunity, penetration, biodistribution, and safety, but none fully predicts clinical efficacy. The schematic should not be interpreted as evidence that current systemic AAV or LNP platforms routinely achieve deep tumor penetration or direct intratumoral T-cell reprogramming. Created by the authors using Microsoft PowerPoint; no previously published material was reproduced or adapted. CAR-T, chimeric antigen receptor T cell; ECM, extracellular matrix; IV, intravenous.

Solid tumors remain the least mature setting for *in vivo* CAR-T therapy. This section therefore treats solid tumors as a set of distinct delivery problems rather than as a single application area. Direct *in vivo* CAR-T generation should be separated from ex vivo CAR-cell therapy; systemic delivery should be separated from intratumoral or regional delivery; T-cell engineering should be separated from macrophage, monocyte, or broader myeloid-cell engineering; and stromal targeting should be separated from direct tumor-antigen targeting (**Figure 2**).

The first distinction is route of administration. Systemic delivery is more clinically scalable, but it must compete with hepatic sequestration, reticuloendothelial clearance, serum protein adsorption, and non-specific uptake before enough vector reaches tumor-associated T cells or tumor beds. Intratumoral or regional delivery may increase local exposure and reduce systemic distribution, but it is less suitable for disseminated or inaccessible disease and does not solve the problem of metastatic heterogeneity. Most current solid-tumor *in vivo* CAR-T evidence should therefore be read in light of route: systemic delivery mainly tests whether vectors can survive whole-body biodistribution, whereas local or regional approaches test whether high local concentration can generate a productive intratumoral immune effect.

The second distinction is where CAR-T cells are generated. Some systems mainly program circulating T cells, which may then need to traffic into tumor tissue. Parayath et al. established this principle using injectable polymer nanocarriers carrying *in vitro*-transcribed CAR or TCR mRNA, which transiently programmed circulating T cells and produced antitumor activity in mouse models including prostate cancer and HBV-related hepatocellular carcinoma (75). This supports *in vivo* T-cell programming without ex vivo manufacturing, but it does not prove efficient direct reprogramming of resident immune cells inside dense tumors. By contrast, claims of intratumoral reprogramming require evidence that CAR-expressing T cells are generated or function locally within tumor tissue, not only that circulating engineered cells can later infiltrate the tumor.

The third distinction is target biology. tyrosinase-related protein 1 (TRP1)-directed CAR-T generation in melanoma represents tumor-antigen targeting. In that model, CD3-targeted ionizable LNPs carrying TRP1 CAR mRNA generated CAR-T cells *in vivo*, supported tumor infiltration, and inhibited TRP1-positive melanoma growth. IL-7 mRNA co-delivery and PD-1 blockade further improved activity, illustrating that solid-tumor *in vivo* CAR-T may need cytokine support or checkpoint modulation to overcome local dysfunction (102). In contrast, FAP-directed strategies primarily target the stromal or fibrotic compartment. Meng et al. used FAP-specific CAR mRNA-LNPs to reprogram host immune cells *in vivo* and target cancer-associated fibroblasts in murine solid-tumor models, with stronger regression when combined with chemotherapy and immune checkpoint blockade (103). Yashaswini et al. used CD5-targeted LNPs carrying anti-FAP CAR mRNA to transiently generate anti-FAP CAR-T cells in a metabolic dysfunction–associated steatohepatitis (MASH) model, reducing fibrosis by depleting profibrogenic hepatic stellate cells and improving liver homeostasis (104). This latter study is informative for fibrotic solid tissue and stromal remodeling, but it is not a tumor-efficacy model.

The fourth distinction is model type and evidentiary strength. Syngeneic mouse models preserve some immune context but often simplify human tumor architecture. Xenografts and patient-derived xenografts can capture human tumor or stromal features but usually lack a fully intact human immune system. Organoid and ex vivo tissue models may help test penetration and antigen heterogeneity, but they do not reproduce systemic biodistribution. Large-animal models are more informative for dose, organ distribution, and safety, but are rarely available for true solid-tumor efficacy. Current solid-tumor *in vivo* CAR-T evidence is therefore best described as preclinical and model-dependent.

Several barriers remain specific to solid tumors. Antigen heterogeneity can permit escape after single-antigen targeting, whereas on-target/off-tumor expression can create toxicity when the target is shared with normal stromal or epithelial compartments. Dense extracellular matrix, abnormal vasculature, high interstitial pressure, hypoxia, and suppressive stromal or myeloid components restrict vector penetration and T-cell function. Hepatic and reticuloendothelial uptake further reduce the fraction of systemically administered vector that productively reaches the tumor. These features mean that systemic targeted AAV or LNP vectors should not be assumed to penetrate dense tumor extracellular matrix or reprogram resident immune populations unless that has been directly demonstrated in the relevant model.

Taken together, current solid-tumor data support feasibility rather than clinical maturity. The strongest evidence is still preclinical and is concentrated around transient mRNA-based delivery, T-cell-targeted nanoparticles, and TRP1 or FAP as proof-of-concept targets. The field has shown that *in vivo* CAR-T can be generated and can function in some solid-tissue settings, but durable clinical translation will require clear separation of route, site of CAR-T generation, target class, engineered cell type, and model system. Solid tumors should therefore be presented as an early proof-of-concept area for *in vivo* CAR-T, not as a validated application.

### 5.3 Autoimmune diseases

Autoimmune disease is not merely another indication added to the same oncology framework. It represents a different pharmacological logic for *in vivo* CAR engineering. In cancer, durable persistence is often viewed as an advantage because continued immune surveillance may suppress minimal residual disease and relapse. In autoimmune disease, by contrast, transient CAR expression is attractive because prolonged depletion of non-malignant immune compartments could produce sustained immunosuppression, prolonged B-cell aplasia, infection risk, and reduced controllability.

**Figure 3:**
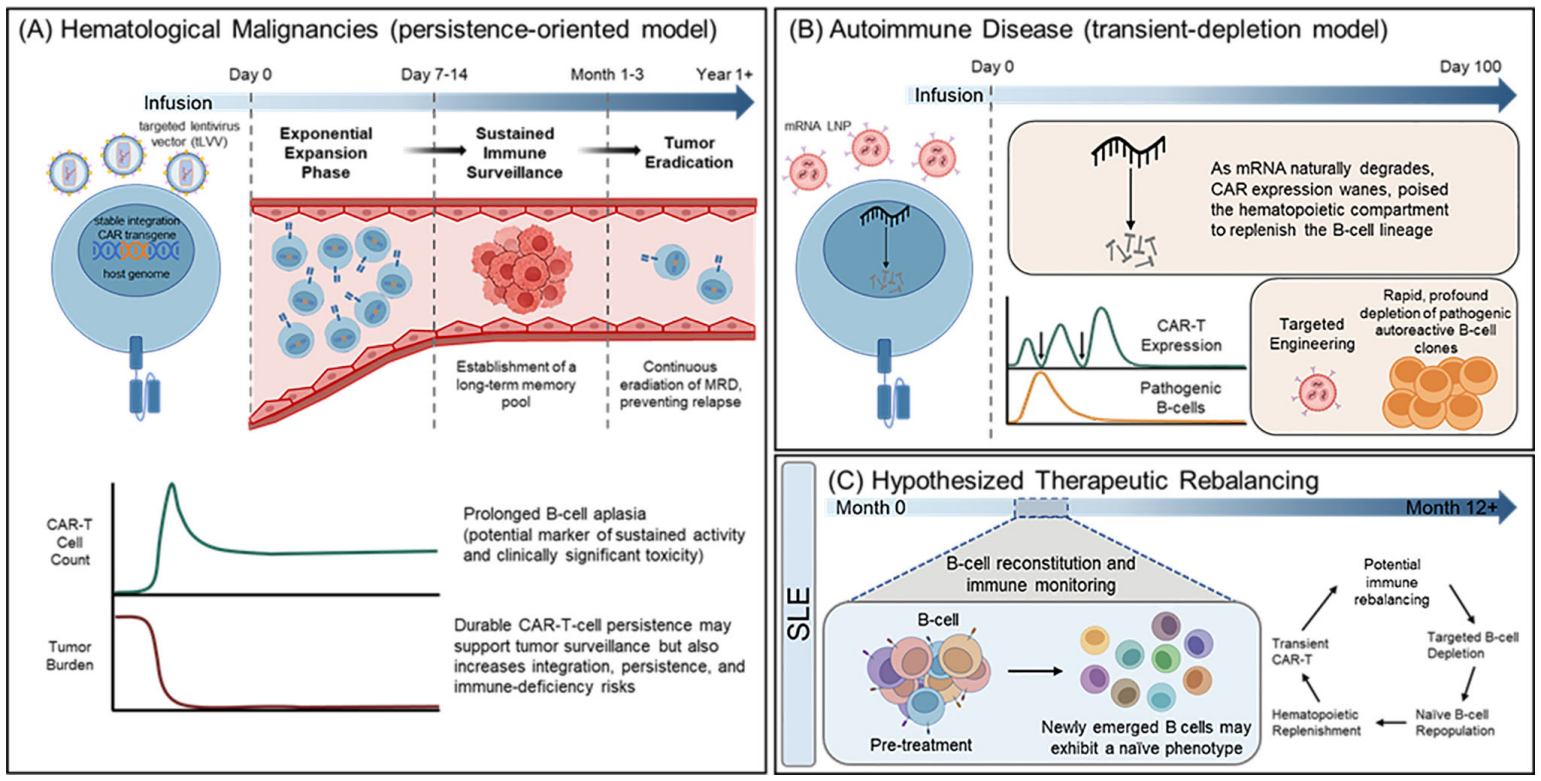
Conceptual longitudinal profiles of *in situ* CAR-T engineering in oncology and autoimmune disease. **(A)** Integrating vectors may support prolonged CAR-T-cell persistence and sustained immune surveillance in hematologic malignancies, but durable expression also raises integration-related risks and may cause prolonged B-cell aplasia, hypogammaglobulinemia, infection, and delayed immune reconstitution. Persistent B-cell aplasia should therefore be interpreted as both a potential pharmacodynamic marker and a clinically relevant toxicity. **(B)** In autoimmune disease, mRNA-LNP delivery may generate a transient pulse of CAR expression and time-limited depletion of pathogenic B-cell compartments, followed by variable B-cell recovery. **(C)** Re-emergence of phenotypically naïve B cells may be consistent with immune rebalancing, but durable immune reset, restored self-tolerance, preserved vaccine responsiveness, and freedom from long-term immunosuppression remain unproven. The curves are conceptual and do not represent validated clinical kinetics. Created by the authors using Microsoft PowerPoint; no previously published material was reproduced or adapted. CAR, chimeric antigen receptor; CAR-T, chimeric antigen receptor T cell; tLVV, targeted lentiviral vector; MRD, minimal residual disease. The curves are conceptual and do not represent validated clinical kinetics.

This therapeutic logic is best framed as an immune-reset hypothesis which focus on depleting disease-driving immune compartments deeply enough to permit less autoreactive immune reconstitution. B-cell depletion, improvement in disease-activity scores, and biomarker changes such as reduced anti-dsDNA antibodies or complement normalization are encouraging early signals, but they are surrogate or short-term observations unless linked to long follow-up, tissue-level depletion, immunoglobulin recovery, vaccine responsiveness, relapse-free reconstitution, and durable clinical remission. However current evidence does not yet prove durable immune resetting or restoration of self-tolerance. The contrasting persistence-oriented and transient-depletion models, together with the hypothesized immune-rebalancing trajectory, are illustrated in **Figure 3**.

This distinction is especially important for targeted LNP/mRNA platforms. Their relatively short expression window may be useful in autoimmune disease if it produces sufficiently deep but reversible depletion. However, the key questions remain unresolved: whether transient CAR expression can eliminate tissue-resident pathogenic B cells as well as peripheral blood B cells; whether CD19-negative plasmablasts and long-lived plasma cells persist as reservoirs of autoantibody production; whether immunoglobulin recovery and vaccine responses are preserved; and whether repeat dosing is feasible without anti-vector immunity, complement activation, or cumulative inflammatory toxicity.

At present, human evidence remains early. HN2301 is the clearest example of an autoimmune-focused *in vivo* CAR program generating early clinical signal rather than established proof of durable disease control. In the currently available reports, this CD8-targeted LNP/mRNA CD19-directed strategy was associated with transient *in vivo* CAR generation, B-cell depletion, reduced disease activity in a small refractory SLE cohort, and biomarker improvement in a subset of patients, including reductions in anti-dsDNA antibodies and normalization of complement in some cases (72). These findings support the plausibility of transient *in vivo* CAR-T for autoimmune disease, but they do not yet establish durable immune resetting, long-term relapse prevention after B-cell reconstitution, preservation of protective humoral immunity, or applicability across autoimmune phenotypes. CPTX2309 extends this same conceptual direction but currently remains best supported by preclinical and translational evidence rather than by mature human efficacy data. In ACR(American College of Rheumatology) Convergence 2024, Capstan disclosure that CPTX2309 can engineer CD8+ T cells from autoimmune-disease patients, including heavily pretreated SLE samples, to express anti-CD19 CAR and rapidly eliminate primary B cells *in vitro (*73). In ACR Convergence 2025, animals administered CPTX2309 by intravenous injection exhibited specific and efficient CAR engineering of CD8+ T cells, leading to rapid and profound B cell depletion in blood and tissues by 3 hours post-treatment in NSG-PBMC mouse model (105). AERA-109 further expands the case for transient CD19-directed B-cell depletion through targeted LNP engineering. Publicly available Aera materials describe mouse and non-human-primate studies in which targeted LNP delivery generated transient anti-CD19 CAR-T cells without preconditioning and produced B-cell depletion across blood and tissue compartments (106). INT2104 and related constructs are important because they represent a different platform logic from the CD8-targeted LNP programs. Here, a CD7-targeted lentiviral system is used to generate CD20-directed CAR-T and CAR-NK cells *in vivo* without lymphodepletion. Reported preclinical data indicate multi-tissue B-cell depletion, peak circulating CAR cells around several weeks after dosing, target-cell killing without direct B-cell transduction (17, 107). UB-VV400 pushes the field further by exploring *in vivo* generation of CD22-directed CAR-T cells using a surface-engineered lentiviral vector with a rapamycin-activated cytokine receptor payload (108). Public sources support both a preclinical abstract describing rapamycin-responsive *in vivo* enrichment and a phase 1 registry record for UB-VV400 in relapsed or refractory B-cell malignancies (108, 109). In the autoimmune context, the importance of this program lies less in immediate disease-specific proof and more in showing that alternative B-cell targets and controllable expansion circuits are technically being explored. The autoimmune setting also broadens the conceptual scope of *in vivo* CAR engineering beyond canonical CD19 depletion.

Even so, several disease-specific uncertainties remain. Autoimmune diseases including systemic sclerosis, inflammatory myopathies, rheumatoid arthritis, or other autoimmune disorders, differ in dominant immune drivers, tissue distribution, autoantibody dependence, fibrotic versus inflammatory pathology, B-cell and plasma-cell contributions, and the clinical meaning of relapse. A depletion strategy that appears promising in refractory SLE may not achieve the same depth, duration, or safety in diseases with more tissue-resident pathology or less clearly B-cell-dominant mechanisms.

Accordingly, autoimmune *in vivo* CAR-T should be described as a promising but still unproven strategy for transient immune depletion or immune rebalancing. However, it cannot yet be concluded that *in vivo* CAR-T has achieved the goal of durable immune reset and restoration of self-tolerance. Future studies will need to report treated-patient numbers, follow-up duration, peripheral and tissue-resident B-cell depletion, CD19-negative plasmablast and long-lived plasma-cell persistence, immunoglobulin recovery, vaccine responses, infection events, antimicrobial prophylaxis, relapse after B-cell reconstitution, retreatment feasibility, anti-vector immunity, and disease-specific outcomes.

## 6 Clinical frontiers and critical challenges

### 6.1 Core challenges limiting clinical translation

A recurring weakness in discussions of *in vivo* CAR-T is that the field’s engineering momentum can obscure how many of its key problems remain translational rather than merely technical. The central issue is not whether CAR cargo can be delivered in principle, but whether it can be delivered reproducibly, selectively, and safely enough to sustain a regulatory-quality therapeutic window across disease settings. Several limitations are especially important in the short-to-medium term.

#### 6.1.1 Technical hurdles: specificity, biodistribution, and deep-tissue delivery

Off-target transduction is not a peripheral concern; it is one of the main factors that may determine whether an *in vivo* CAR platform remains experimentally interesting or becomes clinically usable. Even modest transduction of hepatocytes, macrophages, endothelial cells, or other non-target compartments can simultaneously waste dose, distort pharmacology, and create toxicities that are difficult to interpret because the engineered cells are generated inside the patient rather than under controlled ex vivo release conditions. Early non-targeted LNP studies showed predominant liver delivery after systemic administration, and even targeted systems do not fully eliminate non-specific uptake or ligand-independent adsorption (9, 110). For integrative viral vectors, off-target cell entry creates an additional concern: gene-transfer errors may persist rather than simply wash out.

Uncertain biodistribution compounds this problem. A vector may appear selective in blood or spleen yet behave very differently across marrow, liver, lung, lymphoid tissue, inflamed organs, or tumor beds. This is especially relevant in autoimmune disease, where the target tissue may be distributed and chronically inflamed, and in solid tumors, where anatomical barriers can make systemic exposure a poor surrogate for productive intratumoral engineering (65). As a result, biodistribution is not only a pharmacokinetic parameter but also a major source of uncertainty for dose selection, safety interpretation, and cross-study comparability.

Deep-tissue delivery in solid tumors is a distinct and probably more formidable barrier than blood-borne target access in hematologic disease. Dense extracellular matrix, abnormal vascular permeability, high interstitial pressure, and suppressive stromal or myeloid components all reduce the probability that sufficient vector reaches the right cells in the right anatomical compartment (111). This means that many apparently strong solid-tumor efficacy signals may partly reflect model systems with lower stromal complexity or more permissive exposure conditions than human tumors (112). In practical terms, this delivery problem likely remains one of the main reasons why solid-tumor *in vivo* CAR translation lags behind hematologic applications.

A further platform-wide trade-off concerns persistence versus controllability. Long-lived expression may support durable tumor surveillance, but the same feature can become a liability if toxicity, off-tumor targeting, or unintended immune suppression emerges after administration. Conversely, transient systems such as mRNA-LNPs improve reversibility and redosing flexibility, yet may require repeated dosing to maintain effect, thereby increasing cumulative exposure and potentially compounding systemic toxicity or manufacturing burden. This is not a simple choice between “better” and “worse” platforms; it is a disease-dependent trade-off that must be discussed explicitly rather than assumed away.

#### 6.1.2 Safety concerns: genomic risk, hepatic exposure, and systemic toxicity

Safety evaluation in *in vivo* CAR-T must address both cell-therapy-like risks and vector-specific risks. Acute cytokine release syndrome (CRS) and immune effector cell–associated neurotoxicity syndrome (ICANS) remain important, but they are not the only clinically meaningful hazards (113). For integrating vectors such as targeted lentiviral systems, the possibility of insertional mutagenesis remains a fundamental long-term concern even though modern self-inactivating backbones reduce promoter-mediated risk. The unresolved issue is not whether the risk can be lowered, but whether it can be characterized adequately when gene-modified effector cells are generated directly *in vivo*, potentially across multiple tissues and cell types (114).

For AAV and LNP platforms, the dominant concern shifts from obligatory vector integration toward exposure biology. Recombinant AAV is predominantly episomal and has lower insertional concern than LV. Its limited cargo capacity, pre-existing neutralizing antibodies, rare integration possibility, and dose-dependent hepatic toxicity restrict how broadly its apparent safety can be generalized (115). LNP-based systems avoid viral insertional risk and support redosing, but systemic exposure often remains strongly liver-biased, raising concerns about hepatic accumulation, innate immune activation, and the gap between nominal administered dose and biologically productive dose in target T cells (9). Thus, statements that non-viral systems are simply “safer” than viral systems are often too coarse; they substitute one risk structure for another rather than eliminating risk.

The same caution applies to bioinspired platforms such as extracellular vesicles and related biomimetic carriers. These systems are appealing because of their biocompatibility and potentially lower innate immune activation, yet their safety profile is inseparable from their source, purification method, residual cargo, and batch composition. In other words, what appears biologically elegant at small scale may become far less predictable during scale-up (116).

Because *in vivo* CAR-T creates engineered cells directly inside the patient, safety assessment should be organized around risks that are specific to *in vivo* generation rather than borrowed only from ex vivo CAR-T. **Table 3** summarizes a practical risk matrix linking each risk to recommended preclinical assays and long-term clinical surveillance.

**Table 3:** *In vivo* CAR-specific safety risk matrix.

Risk domain	Mechanistic concern	Most relevant platforms/contexts	Recommended preclinical assays	Clinical surveillance/mitigation
Ectopic CAR expression in non-immune tissues	Vector uptake by hepatocytes, endothelial cells, macrophages, or other non-target tissues may produce CAR expression outside the intended immune compartment	Systemic LNPs, AAV, non-fully retargeted viral vectors, polymeric NPs	Tissue biodistribution; CAR transcript/protein mapping across liver, spleen, marrow, lung, gonad, lymph node, and inflamed tissue	Liver enzymes, inflammatory markers, tissue-specific toxicity panels, vector copy/RNA monitoring in blood and selected tissues when feasible
Accidental transduction of antigen-positive B cells and antigen masking	Delivery of CAR cargo into CD19/CD20/BCMA-positive target cells could mask antigen or alter detectability, creating false-negative target assessment or resistant compartments	B-cell malignancy and autoimmune B-cell depletion programs	In vitro transduction of antigen-positive B cells; antigen-density and CAR-expression co-staining; tumor escape and antigen masking assays	Flow cytometry panels that distinguish antigen loss from CAR-mediated masking; MRD assays
CAR expression in regulatory or immunosuppressive populations	Engineering Tregs or other suppressive populations could blunt efficacy or increase immune suppression	CD3/CD5/CD7/CD8-targeted systems with imperfect subset selectivity	Subset-resolved transduction assays covering CD4, CD8, Treg, exhausted T cells, NK cells, thymocytes, and progenitor-like cells; cytokine and suppressive-function assays	Longitudinal immune phenotyping; Treg/NK subset tracking; functional immune-reconstitution assays
Replication-competent vector risk	Theoretically generate replication-competent vector, even when modern split packaging is used	Lentiviral and other viral-vector platforms	Replication-competent lentivirus/vector testing in vector lots and transduced cells; long culture amplification assays	Lot-release RCL/RCV testing; post-treatment vector monitoring if clinically indicated
Integration-site distribution and insertional oncogenesis	Insert near proto-oncogenes or expand specific clones; SIN design reduces but does not eliminate clonal risk	Lentiviral, AAV vectors	Integration-site analysis; vector copy number; clonal outgrowth assays; genotoxicity and insertion-site mapping in relevant target cells	Long-term follow-up for clonal expansion, hematologic malignancy, unexplained cytopenias, and insertion-site dominance
Long-term vector shedding and biodistribution	Vector genomes or RNA may persist, traffic, or shed beyond the intended exposure window	Viral vectors, AAV, LNPs, polymeric systems	Quantitative vector biodistribution in blood, urine, stool, saliva, semen when relevant; persistence and shedding kinetics	Shedding studies; vector genome/RNA monitoring; precautions for transmissibility only when supported by vector biology
Gonadal exposure and theoretical germline risk	Systemic delivery may expose gonadal tissue even if germline modification is unlikely	AAV, LNPs, lentiviral vectors at systemic doses	Gonadal biodistribution; germ-cell exposure studies in reproductive-toxicology models; vector DNA/RNA detection in reproductive tissues	Pregnancy avoidance windows when justified; reproductive counseling; long-term registry capture for reproductive outcomes
Complement activation and infusion reactions	Nanoparticles, viral capsids, PEG-lipids, or biological membranes can trigger complement activation-related pseudoallergy or acute infusion reactions	LNPs, polymeric NPs, AAV, EV/bioinspired systems	Human serum complement activation assays; cytokine-release assays; infusion-rate and repeat-dose tolerability studies	Monitoring during and after infusion; complement markers, tryptase when indicated, vital signs, premedication or infusion-rate adjustment
Innate immune activation	Activate TLRs, inflammasomes, interferon pathways, or macrophages	LNPs, saRNA/circRNA systems, polymeric NPs, AAV, EVs	Human PBMC cytokine panels; interferon-stimulated gene profiling; inflammasome and complement assays; species-cross-reactive toxicology	Cytokines, fever, inflammatory markers, liver enzymes, repeat-dose immune activation, anti-drug or anti-vector antibody monitoring
CRS and ICANS under uncontrolled in vivo cell generation	The number, phenotype, and expansion kinetics of generated CAR cells cannot be selected or release-tested ex vivo	All active in vivo CAR-T platforms, especially durable viral vectors	In vivo dose-response modeling; CAR-cell expansion kinetics; cytokine-release assays using patient-like immune cells; tumor-burden models	CRS/ICANS monitoring, early intervention, cytokine panels, neurotoxicity assessment, CAR-cell kinetics, inpatient observation for higher-risk settings
Prolonged cytopenias, infection, hypogammaglobulinemia, delayed immune reconstitution	B-cell depletion or broader immune engineering may persist longer than intended, especially with durable vectors or repeat dosing	CD19/CD20/BCMA programs; durable LV/AAV systems; repeat-dose LNP programs	Duration-of-depletion studies; marrow and lymphoid-tissue immune profiling; repeat-dose toxicology	CBC, immunoglobulins, B-cell aplasia duration, infection surveillance, vaccination status, IVIG or antimicrobial support when indicated
Neutralizing antibodies and altered repeat-dose safety	Anti-vector, anti-capsid, anti-PEG, or anti-ligand immunity may change biodistribution, reduce efficacy, or increase toxicity on redosing	AAV, lentiviral vectors, LNPs with PEG/lipid or targeting ligands, bioinspired systems	Repeat-dose immunogenicity; neutralizing-antibody assays; complement and cytokine response after rechallenge	Anti-vector/anti-ligand antibody monitoring; dose-spacing rules; redosing eligibility criteria; alternate-vector strategies
Controllability and stopping mechanisms	Once engineered cells are generated in vivo, stopping or reversing activity may be harder than with ex vivo products selected before infusion	Durable LV/AAV systems; platforms with expansion circuits; high-risk targets	Switch-off systems, suicide genes, pharmacologic enrichment/depletion models, target-antigen withdrawal or rescue studies	Predefined stopping rules; availability of pharmacologic switches or suicide-gene activation if included; monitoring for persistence, off-target toxicity, and delayed immune effects

AAV, adeno-associated virus; BCMA, B-cell maturation antigen; CAR, chimeric antigen receptor; CBC, complete blood count; circRNA, circular RNA; CRS, cytokine release syndrome; DNA, deoxyribonucleic acid; EV, extracellular vesicle; ICANS, immune effector cell-associated neurotoxicity syndrome; IVIG, intravenous immunoglobulin; LNP, lipid nanoparticle; LV, lentiviral vector; MRD, minimal residual disease; NK, natural killer; NP, nanoparticle; PBMC, peripheral blood mononuclear cell; PEG, polyethylene glycol; RCL, replication-competent lentivirus; RCV, replication-competent vector; RNA, ribonucleic acid; saRNA, self-amplifying RNA; SIN, self-inactivating; TLR, Toll-like receptor; Treg, regulatory T cell.

#### 6.1.3 Translational barriers: batch variability, CMC control, and regulatory ambiguity

Some of the hardest barriers to near-term clinical translation are not biological but industrial and regulatory. Extracellular vesicles, erythrocyte-mimetic formulations, and other bioinspired systems face a serious batch-consistency problem: particle composition, cargo loading, membrane protein display, and biological potency may vary materially by source material and process conditions (117). These are not cosmetic CMC issues; they directly affect interpretability, reproducibility, and regulatory confidence.

Even in more established platforms, CMC remains a major bottleneck. Targeted LNPs require reproducible control over ligand density, particle-size distribution, encapsulation efficiency, ionizable lipid behavior, and storage stability. AAV platforms must reconcile manufacturing scalability with the high vector doses required for systemic administration, as prevalent pre-existing capsid immunity and suboptimal transduction efficiency in primary T cells substantially elevate the clinical dosage demand. Targeted lentiviral systems, which rely on engineered surface fusion proteins for cell specificity, require a more complex manufacturing workflow than conventional soluble biologics. Their production process must balance vector potency, consistent ligand display, and comprehensive safety quality characterization. Therefore, it is required not only that a platform can be made, but also that it can be produced with sufficient consistency to support comparability across lots, studies, and clinical sites (118).

Regulatory classification adds a second layer of uncertainty. *In vivo* CAR engineering sits at the boundary between gene therapy and cell therapy: the administered product is a vector, but the pharmacologically active entity is a gene-modified cell population produced inside the patient. This hybrid nature complicates expectations for biodistribution, long-term follow-up, potency assays, release testing, and risk attribution (119). Different agencies may emphasize different elements of the product, creating uncertainty in development strategy and prolonging the path to approval (120, 121). For a field still trying to define clinically meaningful endpoints, that ambiguity is not trivial.

### 6.2 Future directions: actionable translational requirements

Future development should be organized around translational requirements rather than broad enthusiasm for the platform. The key question is no longer whether CAR expression can be induced *in vivo* in selected models, but whether this can be converted into a reproducible, controllable, and regulatory-grade therapeutic process. Progress will require defined benchmarks for delivery specificity, biodistribution, potency, safety, manufacturing comparability, and disease-specific benefit-risk (**Table 4**).

**Table 4:** Actionable translational roadmap for *in vivo* CAR-T development.

Translational requirement	Why it matters	Near-term development benchmark
Selection of the endogenous target-cell receptor	CD3, CD5, CD7, CD8, and other delivery receptors differ in uptake, signaling, subset distribution, and off-target cell coverage	Receptor-by-receptor comparison of uptake, activation, subset engineering, and non-target cell exposure in human cells and relevant animal models
Control of off-target biodistribution	Non-productive uptake in liver, spleen, macrophages, endothelium, or other tissues can reduce efficacy and increase toxicity	Quantitative tissue biodistribution, single-cell transduction mapping, and CAR transcript/protein localization after systemic dosing
Cargo design and expression duration	Durable integration, episomal DNA, mRNA, circRNA create different persistence and controllability profiles	Matched comparison of expression kinetics, CAR-cell expansion, persistence, and reversibility for each cargo format
Quantitative potency assays	In vivo CAR-T products cannot rely only on vector dose; potency depends on generated CAR-cell number, phenotype, and function	Assays linking administered dose to CAR expression, target-cell killing, cytokine release, phenotype, and persistence
Relevant large-animal models	Mouse and humanized-mouse data may not predict human biodistribution, immunogenicity, or target-cell biology	NHP or other large-animal studies where target receptor biology is cross-reactive and tissue distribution can be measured
CMC comparability and batch-release testing	Targeted vectors and nanoparticles may vary in ligand density, particle size, encapsulation, infectivity, potency, and impurity profile	Release assays for identity, purity, ligand display, vector potency, residual impurities, replication-competent vector risk, and batch comparability
Clinical dose escalation and biodistribution monitoring	Dose cannot be interpreted only as vector particles or RNA mass because productive CAR-cell generation may vary across patients	Early trials with dose-escalation rules linked to CAR-cell kinetics, cytokines, biodistribution markers, and on-target depletion
Long-term follow-up for integrating systems	Lentiviral or AAVs require surveillance for integration-site distribution and clonal expansion	Integration-site analysis, vector copy number, clonal dominance monitoring, hematologic surveillance, and long-term genotoxicity follow-up
Repeat-dose strategy for non-integrating systems	LNP, Polymeric, or bioinspired systems may require redosing but can induce anti-vector or anti-ligand immunity	Repeat-dose immunogenicity, neutralizing-antibody monitoring, complement activation testing, and retreatment eligibility criteria
Indication-specific benefit-risk thresholds	Hematologic malignancy, solid tumors, and autoimmune disease require different levels of persistence, depletion depth, reversibility, and acceptable toxicity	Disease-specific target-product profiles defining acceptable persistence, safety monitoring, retreatment logic, and clinical endpoints

AAV, adeno-associated virus; CAR, chimeric antigen receptor; circRNA, circular RNA; CMC, chemistry, manufacturing, and controls; DNA, deoxyribonucleic acid; LNP, lipid nanoparticle; mRNA, messenger RNA; NHP, non-human primate; RNA, ribonucleic acid.

## 7 Conclusion

*In vivo* CAR-T engineering has moved beyond a purely conceptual stage, but it has not yet reached platform-level clinical maturity. The strongest current evidence is uneven: selected hematologic and autoimmune programs now provide early human feasibility signals, whereas solid-tumor evidence remains largely preclinical and model-dependent. This distinction matters because the central question is no longer whether endogenous cells can be engineered *in vivo*. That has been shown in multiple experimental systems. The harder question is whether *in vivo* engineering can be made selective, quantifiable, controllable, manufacturable, and clinically interpretable enough to support reproducible therapeutic development.

A useful development roadmap must therefore begin with the endogenous cell that is being targeted. CD3, CD5, CD7, CD8, and other delivery receptors are not interchangeable address labels (13, 14, 16, 18). They differ in tissue distribution, receptor internalization, signaling biology, subset bias, and risk of engineering non-target populations. The first translational gate is therefore not vector potency alone, but proof that the chosen receptor generates the intended CAR-expressing cell population in humans without unacceptable off-target immune programming. This requirement also separates the delivery receptor from the CAR antigen: a platform that efficiently enters CD8+ T cells and expresses an anti-CD19 CAR must be evaluated both for CD8-targeted delivery biology and for CD19-directed therapeutic effect.

The second gate is biodistribution. For systemic administration, the fraction of vector that reaches productive immune-cell targets may be small relative to liver, spleen, kidneys or other non-target uptake (57, 59, 122). This is not merely a dosing inefficiency; it changes safety interpretation, potency, and cross-patient variability. Solid tumors add an additional barrier because dense extracellular matrix, abnormal vasculature, high interstitial pressure, hypoxia, and antigen heterogeneity may prevent productive intratumoral engineering even when circulating CAR-T generation is demonstrable (69, 102–104). Future studies therefore need quantitative biodistribution, single-cell transduction mapping, and tissue-level CAR expression data, not only systemic vector or peripheral blood readouts.

The third gate is cargo kinetics. Integrating vectors, episomal DNA, linear mRNA, circRNA, transposon systems, and nuclease-assisted designs encode different assumptions about persistence and controllability (10). In hematologic malignancy, durable CAR expression may be useful if it can be balanced against insertional risk, clonal expansion, and long-term immune toxicity (3, 19). In autoimmune disease, the desired profile may be deep but time-limited depletion followed by immune reconstitution; durable immune reset and restored self-tolerance remain hypotheses until supported by longer follow-up, tissue-depletion data, immunoglobulin recovery, vaccine responses, relapse monitoring, and retreatment outcomes (9, 72). In solid tumors, the immediate barrier is often not persistence but physical and immunologic access to the tumor compartment (112).

The fourth gate is measurement. *In vivo* CAR-T cannot be developed with vector dose as the only potency metric. The active product is generated inside the patient, so potency must connect administered dose to the number, phenotype, location, expansion kinetics, and function of the CAR-expressing cells that emerge. A development program will need assays that relate vector identity, ligand display, cargo integrity, transduction efficiency, CAR-cell phenotype, target-cell killing, cytokine release, persistence, and reversibility. Without such quantitative potency assays, comparisons across platforms, lots, doses, and clinical sites will remain difficult to interpret (114, 120).

The fifth gate is translational model relevance and CMC control. Mouse and humanized-mouse studies are useful for mechanisms, but they are often insufficient for predicting human biodistribution, immunogenicity, repeat-dose behavior, or target-cell receptor biology. Relevant large-animal studies will be especially important when receptor binding, organ distribution, and immune recognition are species dependent. In parallel, manufacturing comparability must become part of the scientific argument (58, 120, 121, 123). Ligand density, particle size, vector infectivity, RNA encapsulation, residual impurities, replication-competent vector risk, and lot-to-lot potency are not downstream technicalities; they determine whether the therapy can be regulated as a reproducible product (120).

The final gate is indication-specific benefit-risk, as no current delivery platform is universally optimal. Integrating systems may be appropriate where durable immune surveillance justifies long-term genomic safety and clonal-monitoring requirements, whereas non-integrating RNA-based systems may be preferred when transient expression, dose titration, redosing, and reversibility are priorities (19, 20). However, their clinical value depends on controlling liver-biased biodistribution, dose-dependent cytokine release, innate immune activation, and repeat-dose immunogenicity (59, 61, 62). AAV-based systems, which are predominantly episomal but retain a low integration potential, present a distinct set of constraints, particularly neutralizing-antibody-mediated barriers to redosing and dose-related hepatotoxicity (124, 125). Accordingly, autoimmune diseases, hematologic malignancies, and solid tumors will require different thresholds for persistence, depletion depth, reversibility, toxicity, monitoring, and clinical endpoints.

Thus, the field’s next phase should be judged by expansion of the concept and disciplined translational proof. The decisive studies will be those that select the right endogenous target-cell receptor, show where the vector goes, measure which CAR cells are generated, define expression duration, validate potency, establish CMC comparability, monitor biodistribution during dose escalation, and match risk tolerance to the intended indication. Only when these requirements are met will *in vivo* CAR-T move from promising platform engineering to a clinically dependable therapeutic modality.
